# Serum but not cerebrospinal fluid levels of allantoin are increased in de novo Parkinson’s disease

**DOI:** 10.1038/s41531-023-00505-0

**Published:** 2023-04-12

**Authors:** Lenka Hasíková, Jakub Závada, Tereza Serranová, Petr Kozlík, Květa Kalíková, Lenka Kotačková, Jiří Trnka, David Zogala, Karel Šonka, Evžen Růžička, Petr Dušek

**Affiliations:** 1 Institute of Rheumatology, Prague, Czech Republic; Department of Rheumatology, First Faculty of Medicine, Charles University, Prague, Czech Republic; 2 Department of Neurology and Centre of Clinical Neuroscience, First Faculty of Medicine, Charles University and General University Hospital, Prague, Czech Republic; 3 Department of Analytical Chemistry, Faculty of Science, Charles University, Prague, Czech Republic; 4 Department of Physical and Macromolecular Chemistry, Faculty of Science, Charles University, Prague, Czech Republic; 5 Institute of Medical Biochemistry and Laboratory Diagnostics, First Faculty of Medicine, Charles University and General University Hospital, Prague, Czech Republic; 6 Institute of Nuclear Medicine, First Faculty of Medicine, Charles University and General University Hospital, Prague, Czech Republic; 7 Department of Radiology, First Faculty of Medicine, Charles University and General University Hospital, Prague, Czech Republic

**Keywords:** Parkinson's disease, Neurodegeneration

## Abstract

Oxidative stress supposedly plays a role in the pathogenesis of Parkinson’s disease (PD). Uric acid (UA), a powerful antioxidant, is lowered in PD while allantoin, the oxidation product of UA and known biomarker of oxidative stress, was not systematically studied in PD. We aim to compare serum and cerebrospinal fluid (CSF) levels of UA, allantoin, and allantoin/UA ratio in de novo PD patients and controls, and evaluate their associations with clinical severity and the degree of substantia nigra degeneration in PD. We measured serum and CSF levels of UA, allantoin, and allantoin/UA ratio in 86 PD patients (33 females, mean age 57.9 (SD 12.6) years; CSF levels were assessed in 51 patients) and in 40 controls (19 females, 56.7 (14.1) years). PD patients were examined using Movement Disorder Society-Unified Parkinson’s Disease Rating Scale (MDS-UPDRS), Montreal Cognitive Assessment (MoCA), Scales for Outcomes in Parkinson Disease-Autonomic (SCOPA-AUT), the University of Pennsylvania Smell Identification Test (UPSIT), one-night video-polysomnography, and dopamine transporter single-photon emission computed tomography (DAT-SPECT). Serum allantoin and allantoin/UA ratio were significantly increased in the PD group compared to controls (*p* < 0.001 and *p* = 0.002, respectively). Allantoin/UA ratios in serum and CSF were positively associated with the SCOPA-AUT score (*p* = 0.005 and 0.031, respectively) and RBD presence (*p* = 0.044 and 0.028, respectively). In conclusion, serum allantoin and allantoin/UA ratio are elevated in patients with de novo PD. Allantoin/UA ratio in serum and CSF is associated with autonomic dysfunction and RBD presence, indicating that higher systemic oxidative stress occurs in PD patients with more diffuse neurodegenerative changes.

## Introduction

Parkinson’s disease (PD) is the most common form of neurodegenerative synucleinopathy. The main pathological features of PD are the loss of dopaminergic neurons in substantia nigra pars compacta leading to the depletion of dopamine and the formation of alpha-synuclein positive intracytoplasmatic inclusions referred to as Lewy bodies in surviving neurons^1^. Clinical manifestations of PD are heterogeneous, and several disease phenotypes were defined based on the spectrum of motor and non-motor symptoms. Unbiased clustering analyses showed that patients with dysautonomia and rapid eye movement (REM) sleep behavior disorder (RBD), referred to as the “diffuse malignant” subtype, have a higher risk of cognitive impairment and faster disease progression as compared to patients with only motor involvement, known as the “mild motor-predominant” subtype^2,3^.

There is increasing evidence that implicates oxidative stress as a key driver of the complex mechanism underlying neurodegeneration in PD^4^. Oxidative stress may be involved in the pathogenesis of PD through several mechanisms such as oxidation of dopamine, calcium influx through L-type calcium channels and its associated excitotoxicity, mitochondrial dysfunction, protein misfolding and aggregation, or ferroptosis^5^. Oxidative stress arises when the production of reactive oxygen species (ROS) exceeds clearance by endogenous antioxidants^6^. Uric acid (UA), the end product of purine metabolism in humans, is one of the powerful antioxidants in the extracellular compartment with putative neuroprotective effects. UA is a scavenger of singlet oxygen, peroxy radicals, and hydroxyl radicals^7^. PD is associated with lower serum UA levels, particularly in the later stages of the disease^8^. A series of epidemiological studies suggested that higher levels of UA are associated with a decreased risk of disease development and a slower rate of clinical progression in PD^9,10^. In line with these studies, a recent dose-response meta-analysis reported that higher serum UA levels reduced the risk for PD^11^. Consequently, the effects of UA were studied in preclinical disease models. UA within astrocytes prevented dopaminergic cell death and atrophy induced by oxidative and mitochondrial toxins in cellular PD models^12^. UA showed attenuated toxic effects on dopaminergic cells in a mouse intrastriatal 6-hydroxydopamine model of PD^13^. Because of the evolutionary loss of the functional uricase enzyme, humans are unable to further catabolize UA to the more soluble compound allantoin. However, UA can be nonenzymatically oxidized into allantoin by ROS under conditions of increased oxidative stress^14^. Consequently, allantoin has emerged as a reliable biomarker for assessing the oxidative status both in vitro and in vivo^15^. Whereas serum UA level is influenced by many factors such as diet, renal function, drugs, and variants in the genes coding urate transporters^16^, allantoin and/or allantoin/UA ratio may be more accurate biomarkers compared to UA alone. In contrast to UA, allantoin has been only marginally studied in PD. One study revealed no differences in 24-hour urine excretion of UA, allantoin, and allantoin/UA ratio between PD and healthy subjects^17^. In our previous study, we observed increased serum allantoin and allantoin/UA ratio in patients with isolated RBD (iRBD), which is in most cases the prodromal stage of neurodegenerative synucleinopathies, including PD^18^. This study aimed to compare serum and cerebrospinal fluid (CSF) levels of UA, allantoin, and allantoin/UA ratio in PD and healthy controls, and to evaluate associations of these biochemical parameters with the severity of motor and non-motor symptoms, including the presence of RBD and the degree of substantia nigra degeneration in PD.

## Results

Of 111 patients consecutively included in the BIO-PD study between 11/2015 and 11/2021, 103 had serum samples available in the biobank; 17 samples were excluded due to the use of medication affecting UA levels. Thus, serum samples from 86 patients were analyzed while CSF was available in 51 of them. Serum and CSF samples from 50 controls included between 11/2015 and 06/2021 fulfilling the inclusion criteria were available in our biobank, while 10 samples were excluded due to a history of using medication affecting UA levels, yielding 40 control samples for comparison. Mean freezer storage-times were comparable for PD and control samples (3.3 (SD 1.7) vs 3.1 (SD 1.3) years; *p* = 0.50).

**Fig. 1:**
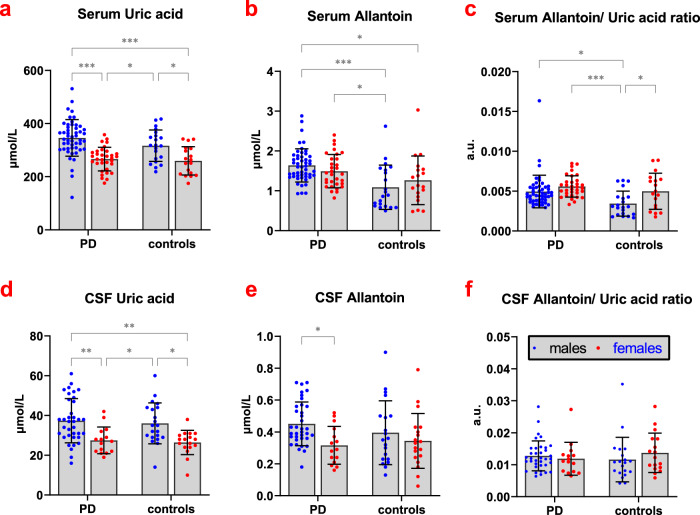
Dot graphs of serum and CSF UA, allantoin, and allantoin/UA ratio (Bars with whiskers represent means and standard deviations). **a** Serum UA, **b** Serum allantoin, **c** Serum allantoin/UA ratio, **d** CSF UA, **e** CSF allantoin, **f** CSF allantoin/UA ratio.

Demographic data of the PD and control groups, as well as results of biochemical analyses are listed in Table 1; The PD subgroup with available CSF had demographic and clinical parameters comparable to the total group (Supplementary Table 1). RBD was diagnosed in 21 (24%, 16 males, 5 females) PD patients. There was a significant effect of sex (*p* < 0.001; higher in men) and BMI (*p* < 0.001; positive association) on serum UA concentrations while no between-group difference was found. Serum allantoin concentrations and serum allantoin/UA ratio were significantly increased in the PD group compared to controls (*p* < 0.001 and *p* = 0.002, respectively); there was a significant effect of storage-time on serum allantoin concentration (*p* = 0.037; positive association) and a significant effect of sex on serum allantoin/UA ratio (*p* = 0.007; higher in women, Fig. 1).

**Table 1:** Demographic data and results of biochemical analyses.

	PD (*n* = 86)	Controls (*n* = 40)	*p* value*
males/females	53/33	21/19	0.340
age [years]	57.9 ± 12.6	56.7 ± 14.1	0.618
disease duration [years]	1.9 ± 1.5	n.a.	n.d.
BMI	27.0 ± 3.5	27.0 ± 5.6	0.982
MDS-UPDRS III	29.4 ± 11.7	n.a.	n.d.
SCOPA-AUT	8.6 ± 5.3	n.a.	n.d.
MoCA	25.1 ± 3.2	n.a.	n.d.
UPSIT	23.4 ± 6.8	n.a.	n.d.
DaTscan (putaminal SBR)	1.6 ± 0.4	n.a.	n.d.
**Serum biochemistry**			
uric acid [μmol/l]^b,s^	Total 314.3 ± 71.8	Total 291.4 ± 63.8	0.088
	Male 346.0 ± 69.0	Male 316.7 ± 59.2	
	Female 266.2 ± 44.4	Female 259.8 ± 53.3	
allantoin [μmol/l]^t^	Total 1.6 ± 0.4	Total 1.2 ± 0.6	**<0.001**
	Male 1.6 ± 0.4	Male 1.1 ± 0.6	
	Female 1.5 ± 0.4	Female 1.3 ± 0.6	
allantoin/ UA ratio^s^	Total 0.005 ± 0.002	Total 0.004 ± 0.002	**0.002**
	Male 0.005 ± 0.002	Male 0.003 ± 0.002	
	Female 0.006 ± 0.001	Female 0.005 ± 0.002	
CSF biochemistry**			
uric acid [μmol/l]^a,s^	Total 34.5 ± 11.0	Total 31.9 ± 9.7	0.269
	Male 37.4 ± 11.1	Male 36.0 ± 10.3	
	Female 27.5 ± 6.7	Female 26.4 ± 6.1	
allantoin [μmol/l]^a,s^	Total 0.4 ± 0.1	Total 0.4 ± 0.2	0.197
	Male 0.5 ± 0.1	Male 0.4 ± 0.2	
	Female 0.3 ± 0.1	Female 0.3 ± 0.2	
allantoin/ uric acid ratio^a^	Total 0.01 ± 0.00	Total 0.01 ± 0.01	0.654
	Male 0.01 ± 0.00	Male 0.01 ± 0.01	
	Female 0.01 ± 0.01	Female 0.01 ± 0.01	

*comparisons of biochemical parameters were adjusted for storage-time, age, sex, and BMI; significant between-group differences are in bold; **CSF was analyzed in 51 PD patients.

^a^significant effect of age (positive association);

^b^significant effect of BMI (positive association);

^s^significant effect of sex (higher in women for serum allantoin/UA ratio; higher in men for all other parameters);

^t^significant effect of storage-time (positive association).

*M* male, *F* female, *PD* Parkinson disease, *BMI* body mass index, *MDS-UPDRS* Movement Disorders Society-Unified Parkinson’s Disease Rating Scale, *SCOPA-AUT* Scales for Outcomes in Parkinson Disease-Autonomic, *MoCA* Montreal Cognitive Assessment, *UPSIT* University of Pennsylvania Smell Identification Test, *SBR* specific biding ratio, *CSF* cerebrospinal fluid, n.a. – not available; n.d. – not done.

CSF UA and allantoin concentrations were significantly correlated with their serum concentrations (*r* = 0.65, *p* < 0.001 and *r* = 0.38, *p* < 0.001, respectively; Supplementary Table 2). There was a significant effect of sex and age on CSF concentrations of UA (*p* < 0.001 with higher concentrations in men and *p* = 0.04, respectively) and allantoin (*p* = 0.03 with higher concentrations in men and *p* < 0.001, respectively) and significant effect of age on CSF allantoin/UA ratio (*p* = 0.01); associations with age were positive for all parameters. No between-group differences in CSF parameters were found.

Adjusted for age and sex, allantoin/ UA ratios in serum and CSF were positively associated with SCOPA-AUT score (*p* = 0.005 and 0.031, respectively) and RBD presence (*p* = 0.044 and 0.028, respectively), whereby the associations with SCOPA-AUT were mostly driven by the gastrointestinal and urinary subscores (Supplementary Table 3). CSF allantoin levels and allantoin/ UA ratio were positively associated with disease duration (*p* < 0.001 and 0.010, respectively). CSF UA levels were positively associated with olfactory function (*p* = 0.013; Table 2). PD patients with RBD had higher allantoin/ UA ratios in the serum (0.006 ± 0.003 vs. 0.005 ± 0.001, *p* = 0.039) and CSF (0.015 ± 0.006 vs. 0.011 ± 0.004, *p* = 0.038) compared to patients without RBD.

**Table 2:** Age and sex-adjusted partial correlations of biochemical and clinical parameters.

	Serum uric acid	Serum allantoin	Serum allantoin/uric acid ratio	CSF uric acid	CSF allantoin	CSF allantoin/uric acid ratio
Disease duration	−0.14	0.11	0.18	0.06	0.50***	0.37**
MDS-UPDRS III	0.02	−0.13	−0.08	−0.05	0.03	0.02
SCOPA-AUT	−0.02	0.25*	0.31**	−0.07	0.23	0.31*
MoCA	0.13	−0.03	−0.12	0.26	0.19	0.01
UPSIT	0.03	0.02	−0.09	0.35*	0.06	−0.27
DaTscan (putaminal SBR)	−0.03	0.05	0.04	−0.04	−0.14	−0.07
RBD	−0.04	0.19	0.23*	−0.16	0.21	0.32*

Partial correlation coefficients adjusted for age and sex together with statistical significance are shown; *p < 0.05; **p < 0.01; ***p < 0.001; significant correlations are in bold.

*MDS-UPDRS* Movement Disorders Society-Unified Parkinson’s Disease Rating Scale, *SCOPA-AUT* Scales for Outcomes in Parkinson Disease-Autonomic, *MoCA* Montreal Cognitive Assessment, *UPSIT* University of Pennsylvania Smell Identification Test, *SBR* specific biding ratio, *RBD REM* sleep behavioral disorder, *CSF* cerebrospinal fluid.

Sensitivity analysis comparing PD and controls excluded due to medications affecting UA levels showed the same pattern of findings as the main analysis. The excluded PD group had higher serum allantoin concentrations (*p* < 0.001) and serum allantoin/UA ratio (*p* < 0.001) compared to the excluded control group; no between-group differences were observed for the CSF parameters (Supplementary Table 4).

## Discussion

Our study investigated the serum and CSF levels of UA, allantoin, and allantoin/UA ratio in patients with PD and their associations with clinical parameters. We found significantly increased levels of serum allantoin and allantoin/UA ratio in PD patients compared to healthy controls, indicating increased oxidative stress. Allantoin/UA ratios in serum and CSF were positively associated with autonomic dysfunction assessed using the SCOPA-AUT questionnaire and with RBD presence. CSF UA levels were positively associated with olfactory function.

Degree of motor and autonomic impairment as well as the prevalence of RBD of our cohort are comparable to previous studies in newly diagnosed PD patients^19,20^ while cognitive performance is similar to the healthy Czech population in this age group^21^, indicating that the patient group is a representative early PD sample.

The determination of allantoin is difficult due to its low concentrations in body fluids, high polarity, and lack of chromophore for UV detection. In previous studies, allantoin was measured in diverse clinical samples, mostly in plasma, with highly variable mean concentrations in healthy controls ranging from 0.9 to 22.0 μmol/l^22^. We employed the UHPLC-MS/MS method using an isotopically labeled internal standard that proved to be precise^23^.

Elevated levels of allantoin, supposedly related to increased systemic oxidative stress, were previously reported in patients with various disorders, including bacterial meningitis, Behçet’s disease, recurrent aphthous stomatitis or chronic renal failure^24,25^. Plasmatic allantoin and allantoin/UA ratio were unrelated to levels of lipid peroxidation product malondialdehyde (MDA)^26^ and may be associated with the protective antioxidant activities under systemic oxidative stress. In line with our results, previous research on oxidative stress markers in PD showed significantly higher levels of blood 8-hydroxyguanosine, nitrite, and MDA and lower levels of catalase, glutathione, and total-cholesterol, indicating the exhaustion of the antioxidative defense system in PD^27^.

In contrast to results from two meta-analyses that revealed lower UA levels in PD patients^8,28^, we did not find a significant difference in serum and CSF UA levels between PD patients and controls. This may be due to the inclusion of newly diagnosed, untreated PD patients - a clinical population that did not show consistent alterations in UA levels in previous studies^29^. Indeed, a meta-analysis confirmed that lower UA levels may be detectable only in later disease stages^8^. Due to its neuroprotective effects, UA was proposed as a potential therapeutic target in PD^12,13^. However, a clinical trial with urate-elevating compound inosine in early PD has not shown any difference in the rate of clinical disease progression over placebo^30^. Moreover, a genome-wide association study of genetic variants underlying urate levels found no causal associations between UA and PD risk, age at onset, or disease progression severity^31^. Hence, UA may not be directly involved in the pathogenesis of PD but rather its decreased levels could represent an epiphenomenon^32^.

In PD patients only one study examining allantoin was conducted until now and no difference between PD and healthy subjects in 24-hour urine excretion of UA, allantoin, and allantoin/UA ratio was found^17^. In contrast to the latter finding, our previous study revealed increased serum allantoin and allantoin/UA ratio in patients with iRBD, implying increased systemic oxidative stress in prodromal synucleinopathy^18^. In the current study, we have also found increased serum allantoin and allantoin/UA ratio in PD, but the effect was smaller, yielding 45% increase in serum allantoin in PD compared to its 86% increase in iRBD males. Although allantoin levels were slightly higher in PD patients with RBD compared to those without RBD, we speculate that a larger increase of allantoin/UA ratio in iRBD compared to PD implies that high serum allantoin in synucleinopathies is most pronounced in the prodromal phase and gradually declines during later disease stages when low UA levels become apparent.

We have observed moderate associations of UA, allantoin or allantoin/UA ratios in serum and CSF with non-motor symptoms, i.e. autonomic and olfactory function and RBD presence. These results are only exploratory and need to be treated with caution since statistical correction for multiple comparisons was not applied. Autonomic dysfunction as well as RBD are associated with a diffuse spread of synuclein pathology in the nervous system, i.e. the diffuse malignant disease subtype^33^. Hence, the increased allantoin/UA ratio may be the systemic reaction to the spread of α-synuclein in the peripheral nervous system^34^. The association with autonomic symptoms were mostly found in the gastrointestinal and urinary domains, which may be related to their early occurrence in synucleinopathies. Interestingly, the association between high allantoin levels and autonomic dysfunction mirrors previous findings related to low UA and non-motor symptoms such as fatigue, attention deficit, anxiety, and cardiovascular disturbances in PD^32,35^, which we, however, could not replicate. We can speculate that higher oxidative stress levels herald a more severe course in the early stage of disease and it may be worth to further study allantoin/UA ratio as a prognostic biomarker in PD.

In our current work, higher UA and allantoin levels in serum and CSF were observed in PD patients taking medication affecting UA levels (mostly thiazide diuretics). In contrast to UA and allantoin levels, allantoin/UA ratio seems to be relatively independent on the use of medication affecting UA in PD and controls. This fact together with its stronger associations with the severity of non-motor symptoms indicates that allantoin/UA ratio may be a more universal and useful marker in synucleinopathies compared to UA or allantoin alone.

We did not find significant between-group differences in allantoin or allantoin/UA ratio in CSF. The fact that between group difference in allantoin and allantoin/UA levels in serum was not paralleled in CSF could reflect a higher extent of oxidative stress on a systemic level possibly reflecting alpha-synuclein spread in the peripheral nervous system in early-stage PD. The antioxidative defense in the CNS may also involve different substances than UA. Despite not being increased, the CSF allantoin/UA ratio was significantly associated with SCOPA-AUT score and RBD status in PD patients. It is, however, not clear whether CSF levels of UA and allantoin are truly related to dysautonomia and RBD, or rather these associations reflect just high cross-correlation between serum and CSF levels of UA and allantoin. This finding is in line with previous reports showing that CSF UA is predominantly determined by serum UA, and is modified by the blood-brain barrier integrity^36^. Yet, under pathological conditions, additional formation of UA from the catabolism of nucleotides, nucleosides, and purine precursors within CNS may occur, as was suggested in patients with bacterial meningitis^37^. Theoretically, allantoin in the CSF could increase at later PD stages with higher oxidative stress in the CNS compartment, which may be supported by the positive association between CSF allantoin and disease duration in our current study.

To our knowledge, this is the first study investigating allantoin and allantoin/UA ratio in serum and CSF in PD patients. The limitation of this study is the relatively small size of the control group. Additionally, only a clinical control group could be used for comparison due to difficulties in obtaining CSF from healthy volunteers. This clinical control group partially consisted of patients with altered CNS functioning, such as sleep disorders, anxiety, or chronic pain, conditions where little is known about oxidative stress involvement. However, the inclusion of heterogeneous control samples from patients with various disorders minimizes the likelihood of significant bias.

In conclusion, we have shown that serum allantoin and allantoin/UA ratio are elevated in patients with de novo untreated PD. Moreover, we found a positive association of allantoin/UA ratio in serum and CSF with autonomic dysfunction and RBD, indicating that allantoin and allantoin/UA ratio may be biomarkers of a more severe PD subtype with accentuated non-motor symptoms.

## Methods

### Participants

This study is part of a longitudinal project “biomarkers in PD (BIO-PD)” aimed at collecting a large representative sample of de novo PD patients^38^. All PD patients were diagnosed based on the Movement Disorder Society clinical diagnostic criteria for PD^39^ and comprehensively examined before the initiation of dopaminergic treatment.

Paired serum/CSF samples from age- and sex-comparable spinal anesthesia subjects and symptomatic controls with noninflammatory, non-neurodegenerative conditions (defined as CSF cell count ≤15 elements/unit of volume and CSF protein concentration ≤1 g/l) were selected from our CSF biobank operated according to Consensus Guidelines for CSF and Blood Biobanking for CNS Biomarker Studies^40^. To minimize potential bias associated with a homogeneous control group, the selected controls included patients with various neurological disorders including functional movement disorder (*n* = 14), narcolepsy (*n* = 3), idiopathic hypersomnia (*n* = 3), headache (*n* = 1), nerve root compression (*n* = 1), anxiety (*n* = 1), spinal stenosis (*n* = 1), and patients undergoing surgery in spinal anesthesia for nonmalignant urologic disorders including prostatic hyperplasia (*n* = 9), hydrocele (*n* = 6), bladder tumor (*n* = 6), urethral stricture (*n* = 3), and phimosis (*n* = 2).

To be eligible for this study, all participants had to be free from medical conditions (i.e. gout, chronic kidney disease, restless legs syndrome, and multiple sclerosis) and medications (i.e, thiazide diuretics or xanthine oxidase inhibitors) that potentially affect UA levels. In controls, a history of neurodegenerative disorders manifesting with a movement disorder or dementia was also an exclusion criterion. Another requirement was the availability of >200 μl serum and (in the case of controls) CSF aliquots stored in the biobank.

This study was approved by the ethics committee of the General University Hospital in Prague. Participants with PD were informed about the study and provided written informed consent; participants from the control group provided samples for biomarker research as per universal biobank informed consent.

### Clinical Examinations

The protocol in PD patients included a structured interview, Movement Disorder Society-Unified Parkinson’s Disease Rating Scale (MDS-UPDRS) part III^41^, Montreal Cognitive Assessment (MoCA)^21^, Scales for Outcomes in Parkinson Disease-Autonomic (SCOPA-AUT) questionnaire^42^, olfactory testing using the University of Pennsylvania Smell Identification Test (UPSIT)^43^, one-night video-polysomnography, and dopamine transporter single-photon emission computed tomography (DAT-SPECT). DAT-SPECT was performed using the [123I]-2-b-carbomethoxy-3b-(4-iodophenyl)-N-(3-fluoropropyl) nortropane ([123I]FP-CIT, DaTscan®, GE Healthcare, Little Chalfont, Buckinghamshire, UK) tracer according to European Association of Nuclear Medicine (EANM) procedure guidelines^44^. The acquisition parameters were as follows: rotational radius 13–15 cm, image matrix 128 × 128, angular sampling with 120 projections at 3° interval and 40 s per view, zoom 1.3, energy window 159 ± 10% keV. Reconstruction of the raw SPECT projection data was performed using the ordered subset expectation maximization (OSEM) algorithm with 8 iterations and 10 subsets including Chang attenuation correction (μ = 0.11 cm^−1^) and 3D Butterworth post-filtering with FWHM = 8 mm^45^. Automated semi-quantitative analysis was performed using the BasGan V2 software and specific binding ratios (SBR) in both putamina were calculated according to the formula [(putamen binding–background binding)/background binding]; the lower value from both hemispheres was used for further analyses^46^. RBD was diagnosed based on clinical history and video-polysomnography according to the International Classification of Sleep Disorders, third edition (ICSD-3)^47^.

### Laboratory analyses

In PD patients, venous blood and optionally CSF samples were drawn at the baseline visit in the morning after an overnight fast. In controls, sampling was performed in the morning after an overnight fast either at the neurology or urology department. Lumbar punctures were performed using 22 G atraumatic needles in PD patients and neurologic controls and by 25 G needles in patients undergoing spinal anesthesia.

Sera were separated within 60 minutes after collection and then aliquoted. CSF was immediately centrifuged at 3000 rpm under 4 °C, and the supernatant was aliquoted. All samples were frozen at −80 °C until further biochemical analysis.

The concentration of UA in serum and CSF was determined using the Beckman Coulter AU system with Beckman Coulter Uric Acid kits (Beckman Coulter, Brea, CA, USA) in a standard biochemical laboratory at the Institute of Rheumatology in Prague.

Allantoin concentration in serum and CSF was determined by ultra-high performance liquid chromatography-tandem mass spectrometry (UHPLC-MS/MS) with an isotopically labeled internal standard. Before the analysis, protein precipitation was used. Samples were processed as follows: 60 µL of 100% acetonitrile (containing 1.33 μM allantoin-^13^C_2_, ^15^N_4_ as an internal standard) was added to 20 µL of the sample. The mixture was vortexed and centrifuged at 16500×g for 6 min. 50 µL of supernatant was transferred into an LC vial. For the UHPLC-MS/MS analysis, a UHPLC system Agilent 1290 with a Triple Quad 6460 mass spectrometer (Agilent Technologies, Waldbronn, Germany) was used. The hydrophilic interaction liquid chromatography (HILIC) conditions consisted of the Acquity BEH Amide column; isocratic elution by a mobile phase composed of acetonitrile and 0.1% formic acid 90/10 (v/v). The flow rate of the mobile phase was maintained at 0.3 mL/min, and the injection volume was 2 µL. The temperature of the column was kept at 30 °C and samples were thermostated at 10 °C. The MS/MS spectrometer was operated in a positive mode. The applied conditions of the electrospray ion source were as follows: gas temperature: 300 °C, gas flow: 7 L/min, sheath gas temperature: 300 °C, sheath gas flow: 8 L/min, nebulizer pressure: 310 kPa and capillary voltage: 6000 V. The MS/MS measurement was performed in multiple reaction-monitoring mode (MRM). Two MRM transitions were monitored for allantoin: Quantifier transition was 159 > 116 (collision energy 2 V and fragmentor voltage 50 V) and a qualifier transition was 159 > 99 (collision energy 8 V and fragmentor voltage 50 V). The transition 165 > 120 (collision energy 2 V and fragmentor voltage 50 V) was monitored for allantoin-^13^C_2_, ^15^N_4_.This method, which has been validated in terms of linearity, lower limit of quantification (LLOQ), upper limit of quantification (ULOQ), accuracy, precision, selectivity, recovery, carry-over effect, matrix effects, robustness, and stability of both quality control (QC) samples and clinical patient samples, was demonstrated to be suitable for its intended purpose. The UHPLC-MS/MS method with one-step sample preparation provided high sensitivity and high sample throughput^23^.

### Statistical analyses

Comparisons of UA, allantoin, and allantoin/UA ratio in the serum and CSF between PD and controls were performed using a univariate general linear model with group and sex as fixed factors, and with age, body mass index (BMI), and freezer storage-time as covariates. Difference in sample storage-times may influence the concentration of biomolecules and was thus suggested to be included as a covariate in longitudinal cohort studies^48^. Post-hoc multiple comparisons were performed using Tukey’s HSD. Chi-square statistics were used to analyze binary variables. Pearson partial correlation coefficients controlling for age and sex were calculated to examine relationships between biochemical and clinical variables. Correlation analyses were considered exploratory and corrections for multiple comparisons were thus not applied. IBM SPSS statistics version 25 (IBM, Armonk, NY, USA) was used for statistical analysis; graphs were plotted using Graphpad Prism version 9.0 (Graphpad software, San Diego, CA, USA).

### Reporting summary

Further information on research design is available in the Nature Research Reporting Summary linked to this article.

## Supplementary information

Supplementary Table 1, Table 2, Table 3, Table 4

reporting summary

## Data Availability

Individual participant data that underlie the findings of this study are available upon request to the corresponding author by qualified researchers (i.e., affiliated to a respected university or research institution/hospital).

## Code availability

Not relevant.
