# Supplementary material for: Serum but not cerebrospinal fluid levels of allantoin are increased in de novo Parkinson’s disease
Source: NPJ Parkinsons Dis. 2023 Apr 12;9:60. doi: 10.1038/s41531-023-00505-0 (PMC10097817; doi:10.1038/s41531-023-00505-0)
Supplement: Supplementary file 1 — Supplementary Table 1, Table 2, Table 3, Table 4 [file 41531_2023_505_MOESM1_ESM.pdf]

**Supplementary Table 1** Demographic data of PD patients with available CSF		
	Total PD (n=86)	PD with CSF (n=51)
males/ females	53/ 33	36/15
age [years]	57.9 ± 12.6	55.2 ± 11.8
disease duration [years]	1.9 ± 1.5	1.8 ± 1.3
BMI	27.0 ± 3.5	26.7 ± 2.8
MDS-UPDRS III	29.4 ± 11.7	28.0 ± 11.1
SCOPA-AUT	8.6 ± 5.3	7.9 ± 4.8
MoCA	25.1 ± 3.2	25.2 ± 3.3
UPSIT	23.4 ± 6.8	24.5 ± 6.6
DaTscan (putaminal SBR)	1.6 ± 0.4	1.6 ± 0.3

Supplementary Table 2 Cross-correlation matrix of biochemical parameters

	serum UA	serum allantoin	serum allantoin/UA ratio	CSF UA	CSF allantoin	CSF allantoin/UA ratio
serum UA	-	0.34***	-0.35***	0.65***	0.34**	-0.19
serum allantoin	-	-	0.70***	0.15	0.38***	0.22*
serum allantoin/UA ratio	-	-	-	-0.26*	0.17	0.38***
CSF UA	-	-	-	-	0.44***	-0.33**
CSF allantoin	-	-	-	-	-	0.63***
CSF allantoin/UA ratio	-	-	-	-	-	-

Pearson correlation coefficients with statistical significance are shown;* p < 0.05;
** p < 0.01; *** p < 0.001; n.s. – not significant
CSF – cerebrospinal fluid; UA – uric acid

Supplementary Table 3 Age and sex adjusted partial correlations of biochemical parameters and SCOPA-AUT subscores

	Serum uric acid	Serum allantoin	Serum allantoin/uric acid ratio	CSF uric acid	CSF allantoin	CSF allantoin/uric acid ratio
Gastrointestinal	-0.03	0.19	0.21	0.01	**0.36***	**0.31***

Urinary	-0.06	0.21	**0.31****	-0.13	0.14	**0.29***
Cardiovascular	0.00	0.07	0.07	0.03	-0.26	-0.12
Pupillomotor	-0.03	-0.05	0.07	0.03	0.11	0.14
Thermoregulatory	0.05	0.17	0.11	0.15	0.24	0.06
Sexual	0.03	0.14	0.17	-0.26	-0.07	0.17

Partial correlation coefficients adjusted for age and sex together with statistical significance are shown; * $p < 0.05$; ** $p < 0.01$; significant correlations are in **bold**

SCOPA-AUT - Scales for Outcomes in Parkinson Disease-Autonomic; CSF – cerebrospinal fluid

Supplementary Table 4 Analysis of excluded subjects

	PD (n=17)	Controls (n=10)	p-value*
males/ females	11/ 6	10/ 0	0.033
age	68.6 ± 8.7	65.7 ± 8.7	0.413
BMI	30.0 ± 4.7	31.3 ± 5.7	0.557
medication (thiazide diuretics/ allopurinol)	15/4	8/5	n.d.
**Serum biochemistry**			
uric acid	404.3 ± 83.6	433.2 ± 108.9	0.644
allantoin [a]	2.2 ± 0.8	1.2 ± 0.5	**<0.001**
allantoin/ uric acid ratio [a,t]	0.006 ± 0.002	0.003 ± 0.001	**<0.001**
**CSF biochemistry****			
uric acid	48.8 ± 20.9	53.1 ± 19.2	0.947
allantoin	0.5 ± 0.2	0.7 ± 0.1	0.409
allantoin/ uric acid ratio	0.01 ± 0.00	0.01 ± 0.01	0.494

* comparisons of biochemical parameters were adjusted for age, sex, and storage time; significant between-group differences are in **bold**; ** CSF was analyzed in 8 PD patients; [a] significant effect of age (positive association); [t] significant effect of storage time (positive association)

PD – Parkinson disease; BMI – body mass index; n.d. – not done
