# Supplementary material for: Serum but not cerebrospinal fluid levels of allantoin are increased in de novo Parkinson’s disease
Source: NPJ Parkinsons Dis. 2023 Apr 12;9:60. doi: 10.1038/s41531-023-00505-0 (PMC10097817; doi:10.1038/s41531-023-00505-0)
Supplement: Supplementary file 2 — reporting summary [file 41531_2023_505_MOESM2_ESM.pdf]

Corresponding author(s): Petr Dusek

Last updated by author(s): Mar 22, 2023

Reporting Summary

Nature Portfolio wishes to improve the reproducibility of the work that we publish. This form provides structure for consistency and transparency in reporting. For further information on Nature Portfolio policies, see our Editorial Policies and the Editorial Policy Checklist.

Statistics

For all statistical analyses, confirm that the following items are present in the figure legend, table legend, main text, or Methods section.

n/a	Confirmed	
☐	☒	The exact sample size (*n*) for each experimental group/condition, given as a discrete number and unit of measurement
☒	☐	A statement on whether measurements were taken from distinct samples or whether the same sample was measured repeatedly
☐	☒	The statistical test(s) used AND whether they are one- or two-sided<br>*Only common tests should be described solely by name; describe more complex techniques in the Methods section.*
☐	☒	A description of all covariates tested
☐	☒	A description of any assumptions or corrections, such as tests of normality and adjustment for multiple comparisons
☐	☒	A full description of the statistical parameters including central tendency (e.g. means) or other basic estimates (e.g. regression coefficient) AND variation (e.g. standard deviation) or associated estimates of uncertainty (e.g. confidence intervals)
☐	☒	For null hypothesis testing, the test statistic (e.g. *F*, *t*, *r*) with confidence intervals, effect sizes, degrees of freedom and *P* value noted<br>*Give P values as exact values whenever suitable.*
☒	☐	For Bayesian analysis, information on the choice of priors and Markov chain Monte Carlo settings
☒	☐	For hierarchical and complex designs, identification of the appropriate level for tests and full reporting of outcomes
☐	☒	Estimates of effect sizes (e.g. Cohen's *d*, Pearson's *r*), indicating how they were calculated

Our web collection on statistics for biologists contains articles on many of the points above.

Software and code

Policy information about availability of computer code

Data collection	no software was used
Data analysis	IBM SPSS statistics version 25

For manuscripts utilizing custom algorithms or software that are central to the research but not yet described in published literature, software must be made available to editors and reviewers. We strongly encourage code deposition in a community repository (e.g. GitHub). See the Nature Portfolio guidelines for submitting code & software for further information.

Data

Policy information about availability of data

All manuscripts must include a data availability statement. This statement should provide the following information, where applicable:

- Accession codes, unique identifiers, or web links for publicly available datasets
- A description of any restrictions on data availability
- For clinical datasets or third party data, please ensure that the statement adheres to our policy

Individual participant data that underlie the findings of this study are available upon request to the corresponding author by qualified researchers (i.e., affiliated to a respected university or research institution/hospital).

Human research participants

Policy information about studies involving human research participants and Sex and Gender in Research.

Reporting on sex and gender	In all statistical analyses of biochemical parameters, sex was used as a fixed factor. Results are reported for the total (aggregate) group as well as for males and females separately. Sex was considered as a binary variable and was self-reported by participants.
Population characteristics	86 (53 males, 33 females) newly diagnosed treatment-naive Parkinson disease patients with a mean age 57.9 (SD 12.6) years and mean motor symptom duration 1.9 (SD 1.5) years.<br>40 (21 males, 19 females) controls in whome spinal tap was perfomed, consisting in spinal anesthesia subjects and symptomatic controls with non-inflammatory, non-neurodegenerative conditions with a mean age 56.7 (SD 14.1) years.
Recruitment	All patients with newly diagnosed untreated Parkinson disease referred to the Movement Disorders Center, Department of Neurology, First Faculty of Medicine, Charles University and General University Hospital (Prague, Czechia) were offered to participate in the longitudinal BIO-PD study. It can be expected that younger and more physically fit patients would agree with participation; results threoretically may not be fully generalizable to older patients with more physical disability. Controls samples were obtained from patients undergoing CSF tap at the department of neurology for diagnostic reasons or spinal anesthesia for urologic surgery. This clinical control group partially consisted of patients with altered CNS functioning, such as sleep disorders, anxiety, or chronic pain, conditions where little is known about their influence on uric acid metabolism. However, the inclusion of heterogeneous control samples from patients with various disorders minimizes the likelihood of significant bias.
Ethics oversight	Ethics committee of the General University Hospital in Prague

Note that full information on the approval of the study protocol must also be provided in the manuscript.

Field-specific reporting

Please select the one below that is the best fit for your research. If you are not sure, read the appropriate sections before making your selection.

☒ Life sciences ☐ Behavioural & social sciences ☐ Ecological, evolutionary & environmental sciences

For a reference copy of the document with all sections, see nature.com/documents/nr-reporting-summary-flat.pdf

Life sciences study design

All studies must disclose on these points even when the disclosure is negative.

Sample size	No sample-size calculation was performed. All available samples stored at our biobank fullfilling inclusion criteria were analyzed. The rationale behind the sufficiency of the sample size comes from our previous study where we have documented significant, almost 90%, increase of serum allantoin in a group of 38 patients with prodromal Parkinson disease when compared to 47 controls (DOI: 10.1016/j.parkreldis.2021.07.031).
Data exclusions	Pre-established exclusion criteria for all participants were medical conditions and medication that potentially affect uric acid levels (i.e. gout, chronic kidney disease, restless legs syndrome, multiple sclerosis, thiazide diuretics, and xanthine oxidase inhibitors). In controls, a history of neurodegenerative disorders manifesting with a movement disorder or dementia as well as abnormal CSF finding (defined as CSF cell count ≤15 elements/unit of volume and CSF protein concentration ≤1 g/l) were also exclusion criteria.
Replication	Employed biochemical methods were previously shown to be stable and reliable. No replication measurements were thus performed.
Randomization	N/A - not an interventional trial
Blinding	Investigators performing biochemical analyses of samples were blinded to clinical data. Neurologist performing clinical examinations was attending physian of included patients and was, thus, not blinded to their clinical status.

Reporting for specific materials, systems and methods

We require information from authors about some types of materials, experimental systems and methods used in many studies. Here, indicate whether each material, system or method listed is relevant to your study. If you are not sure if a list item applies to your research, read the appropriate section before selecting a response.

Materials & experimental systems

n/a	Involved in the study
☒	☐ Antibodies
☒	☐ Eukaryotic cell lines
☒	☐ Palaeontology and archaeology
☒	☐ Animals and other organisms
☐	☒ Clinical data
☒	☐ Dual use research of concern

Methods

n/a	Involved in the study
☒	☐ ChIP-seq
☒	☐ Flow cytometry
☒	☐ MRI-based neuroimaging

Clinical data

Policy information about clinical studies

All manuscripts should comply with the ICMJE guidelines for publication of clinical research and a completed CONSORT checklist must be included with all submissions.

Clinical trial registration: N/A - not an interventional trial

Study protocol: The full description of the BIO-PD study protocol was published previously (DOI: 10.48095/cccsnn2020633)

Data collection: Parkinson disease patients were consecutively included at the Department of Neurology, First Faculty of Medicine, Charles University and General University Hospital (Prague, Czechia) between November 2015 and November 2021.
Control samples were collected at the Department of Neurology or Department of Urology, First Faculty of Medicine, Charles University and General University Hospital (Prague, Czechia) between November 2015 and June 2021.

Outcomes: N/A - not an interventional trial
